# Epidemiology and Genetic Diversity of *Chlamydia pecorum* in Cattle and Sheep from Western China

**DOI:** 10.3390/pathogens14121209

**Published:** 2025-11-27

**Authors:** Mengtao Zhang, Wen Wang, Daqin Xu, Xincheng Qin, Junrong Liang, Bangcheng Guo, Zhen Zhu, Zhongqiu Teng, Nan Bai, Binguo Rong, Jia He, Lupeng Dai, Xue Zhang, Tian Qin

**Affiliations:** 1 Department of Epidemiology and Statistics, College of Public Health, Zhengzhou University, Zhengzhou 450001, China; zmt5190@163.com; 2 National Key Laboratory of Intelligent Tracking and Forecasting for Infectious Diseases, National Institute for Communicable Disease Control and Prevention, Chinese Center for Disease Control and Prevention, Beijing 102206, China; wangwen@icdc.cn (W.W.); qinxincheng@icdc.cn (X.Q.); liangjunrong@icdc.cn (J.L.); tengzhongqiu@icdc.cn (Z.T.); hejia@icdc.cn (J.H.); dailupeng1995@163.com (L.D.); zx1127817@163.com (X.Z.); 3 Gansu Provincial Center for Disease Control and Prevention, Lanzhou 730070, China; xudaqin123@sina.com; 4 Ningxia Center for Disease Control and Prevention, Yinchuan 750000, China; guobangcheng123@sina.com (B.G.); bainan757223@sina.com (N.B.); 5 School of Life Sciences and Food Engineering, Hebei University of Engineering, Handan 056038, China; zhuzhen234@yeah.net; 6 Zhangye Center for Disease Control and Prevention, Zhangye 734000, China; rongbinguo932287@sina.com

**Keywords:** *Chlamydia pecorum*, zoonosis, *ompA* genotyping, MLST, genetic diversity

## Abstract

*Chlamydia pecorum* is a widespread zoonotic pathogen infecting livestock and wildlife, with recent reports of severe human infection. To assess its epidemiological threat, we investigated its prevalence, genetic diversity, and evolutionary dynamics in livestock from western China. Rectal swabs (*n* = 1322) were collected from cattle and sheep across four provinces in western China in 2024–2025. Samples were screened by Nested PCR, and positives were characterized by *ompA* genotyping and multilocus sequence typing (MLST). Overall, 18.9% of samples tested positive for *C. pecorum*. *ompA* analysis defined 33 sequence similarity-based clades (17 unique to the region), while MLST revealed 114 sequence types (111 novel). Discordance between *ompA* and MLST trees highlighted recombination and complex evolutionary trajectories. These findings demonstrate both a high prevalence and marked genetic heterogeneity of *C. pecorum* in western Chinese livestock, with numerous unique local clades and sequence types highlighting its ongoing evolution and zoonotic potential. Therefore, this study provides a foundational genetic database and has prompted the creation of a One Health surveillance network, which are essential for precise source-tracing and early detection to mitigate zoonotic spillover risk.

## 1. Introduction

The Chlamydiaceae family comprises a single genus, *Chlamydia*, which includes fourteen recognized species and several Candidatus species of Gram-negative, obligate intracellular bacteria with a biphasic developmental cycle [1]. Members of this family are globally distributed and infect over 400 host species, ranging from wildlife and companion animals, to humans [2]. While the pathogenic potential of many species remains unclear, several *Chlamydia* spp. are well-established threats to both human and animal health [3]. The most significant species include zoonotic pathogens such as avian *C. psittaci* and ovine enzootic *C. abortus*, along with the ubiquitous livestock-associated *C. pecorum* [4].

*C. pecorum* was recognized as the fourth species of the genus *Chlamydia* in 1992, following its taxonomic separation from *C. psittaci* [5]. It displays a broad host range, predominantly infecting livestock such as cattle, sheep, goats, and pigs, but it has also been detected in wild ruminants, wild boar, small marsupials, and birds [6]. In animals, *C. pecorum* is associated with a wide spectrum of clinical conditions, including poly-arthritis, encephalomyelitis, enteritis, pneumonia, endometritis, vaginitis, and abortion [7]. Notably, two recent cases of community-acquired pneumonia (CAP) in humans caused by *C. pecorum* have been documented in China. The first case involved severe CAP complicated by respiratory failure [8], while the second patient developed severe CAP with respiratory failure, shock, and acute kidney injury, ultimately resulting in death following rapid clinical deterioration [9]. Two patients were diagnosed with *C. pecorum* infection via metagenomic next-generation sequencing (mNGS), both reporting a history of livestock exposure. *C. pecorum* can be transmitted from livestock to humans, posing a zoonotic potential risk.

The *ompA* gene has been widely employed for genotyping *C. pecorum*, with studies collectively identifying 15 *ompA* genotypes (A-O) that segregate into two distinct clades [10]. However, due to frequent mutation and recombination in the *ompA* gene of *C. pecorum*, its sequence exhibits high variability, making it difficult to accurately reconstruct the true phylogenetic relationships of *C. pecorum* [11]. This limitation has driven the adoption of multilocus sequence typing (MLST), which analyzes seven housekeeping genes of *C. pecorum* to determine its sequence types, thereby achieving more precise localization in disease traceability analysis [12]. A strategy combining *ompA* with MLST has been widely adopted, enabling more precise identification of the strains responsible for infections and offering distinct advantages in traceability analysis. For example, in an Australian case of ovine abortion, MLST successfully identified ST23 as the causative strain, while a study in Switzerland utilizing both *ompA* and MLST revealed high genetic diversity among local *C. pecorum* isolates [6,13].

Previous studies have indicated a high infection rate of *C. pecorum* in China, particularly in the pastoral regions of western China, where the prevalence in livestock is significantly higher than in other areas [14]. However, most previous investigations have relied on serological methods for prevalence surveys, which may not accurately reflect the true infection status and fail to establish reliable phylogenetic relationships with globally circulating *C. pecorum* strains. Furthermore, accumulating evidence suggests that *C. pecorum* may possess a greater zoonotic potential than previously recognized [4]. This knowledge gap hampers the ability of healthcare professionals to rapidly identify and manage human infections, particularly within a One Health framework. To address this limitation, we investigated the prevalence and genetic diversity of *C. pecorum* in western China, a key livestock-producing region. Our findings aim to raise awareness of its zoonotic risk and strengthen traceability systems for future outbreak investigations.

## 2. Method

### 2.1. Sample Collection

**Figure 1:**
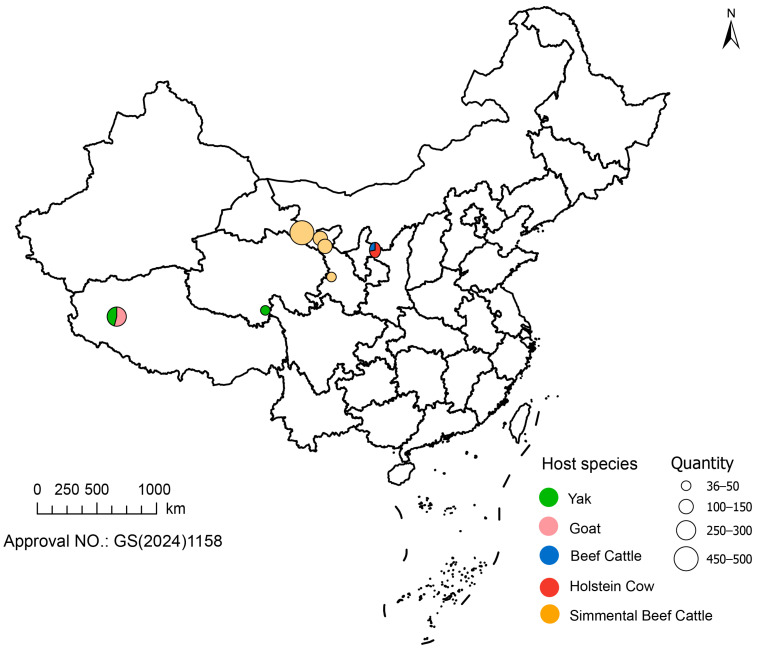
Sampling locations and sample sizes of livestock species in Northwest China. The map shows the geographical distribution of 1322 samples across Gansu, Qinghai, Ningxia, and Tibet. The color of each circle corresponds to a specific species, and the size corresponds to the number of samples collected. The standard map of China was retrieved from https://geo.datav.aliyun.com/areas_v3/bound/100000_full.json (accessed on 5 November 2025). The map approval number is GS (2024)1158. It was constructed using ArcGIS Pro 3.5.2 software.

In this study, given that the first case was detected in Gansu Province, we initially selected four regions with extensive pastoral distribution within the province along a north–south transect. Based on livestock breeding density, 1–3 farms were chosen from each region, and a total of 706 samples were collected. Subsequently, considering the developed livestock industry in Ningxia Hui Autonomous Region, we selected four large-scale farms in Wuzhong City and collected an additional 298 samples [15]. Furthermore, based on existing collaborations, we included 50 bovine swab samples from the Yushu region of Qinghai Province, as well as 145 sheep swab samples and 123 yak swab samples from the Ngari region of Tibet Autonomous Region. During the sampling process, swabs were collected from 3–5 spatially separated locations within each farm, and only healthy animals were selected to minimize selection bias. The detailed sampling distribution is provided in Figure 1.

Sterile cotton swabs were used to obtain rectal contents, which were immediately preserved in sucrose–phosphate–glutamate (SPG) buffer and maintained at 4 °C during transportation. All samples were transported under cold-chain conditions and delivered to the laboratory within 24 h. Upon arrival, specimens were stored at −80 °C in an ultra-low temperature freezer until further processing.

### 2.2. Nucleic Acid Extraction and Concentration

Frozen samples were thawed on ice, and 2 mL of suspension was transferred into 1.5 mL centrifuge tubes. Centrifugation was carried out at 14,000× *g* for 30 min at 4 °C, (Eppendor, Germany) after which the supernatant was discarded and the pellet retained.

Genomic DNA was extracted using the QIAamp PowerFecal Pro DNA Kit (Qiagen, Hilden, Germany) according to the manufacturer’s instructions. DNA was eluted in 70 μL of buffer and stored at 4 °C until use.

### 2.3. C. pecorum ompA Genotyping

To investigate the prevalence and genetic diversity of *Chlamydia*, a nested PCR assay was designed to amplify the variable domains (VDs) of the *ompA* gene, generating an 800 bp product [16] (primer sequences are provided in Supplementary Materials S1). A total of 250 *C. pecorum* positive samples were identified. Resulting sequences were assembled and trimmed using BIOEdit (Ibis Biosciences, Carlsbad, CA, USA) and subsequently deposited in GenBank (Supplementary Materials S2).

For *ompA*-based analysis, 132 sequences obtained in this study were combined with 74 reference sequences from GenBank. Neighbor-joining (NJ) phylogenetic trees were constructed using the Kimura 2-parameter model in MEGA11 (Molecular Evolutionary Genetics Analysis, version 11) [17], with bootstrap support calculated from 1000 replicates. Given the high genetic diversity of *C. pecorum*, sequences were grouped into 33 distinct clades based on a sequence identity threshold of ≥96%. To further assess genetic divergence among these clades, a representative strain from each was selected, and its *ompA* sequence was analyzed using TBtools-II to evaluate sequence homology and generate a heatmap [18].

### 2.4. Multilocus Sequence Typing

To characterize the genetic diversity of *C. pecorum* in China, we applied the internationally standardized MLST scheme [12]. Specific primers were designed to amplify seven housekeeping genes. Consensus sequences were trimmed to standardized lengths after alignment with reference sequences in the Chlamydiales PubMLST database (http://pubmlst.org/chlamydiales/, accessed on 15 August 2025) [19]. A concatenated sequence of 3095 bp was generated by aligning the seven loci in the order gatA-oppA-hflX-gidA-enoA-hemN-fumX. This dataset integrated 182 *C. pecorum* MLST sequences obtained in this study with 101 reference sequences from the Chlamydiales PubMLST database. NJ phylogenetic trees were reconstructed using the Kimura 2-parameter model in MEGA11 [17], with bootstrap support calculated from 1000 replicates.

To investigate the global dissemination of *C. pecorum*, 114 sequence types (STs) identified in China were compared with 89 STs retrieved from the Chlamydiales PubMLST database. Phylogenetic relationships among STs were inferred using the goeBURST algorithm implemented in PHYLOViZ (version 2.0), with a double-locus variant (DLV) threshold applied to define connections [20,21]. This approach enabled visualization of evolutionary linkages and delineation of clonal complexes (CCs) based on allelic differences between strains.

### 2.5. Comparison of MLST and ompA Phylogenetic Analyses in C. pecorum

To assess the congruence between *ompA* and MLST in resolving the evolutionary relationships of *C. pecorum*, phylogenetic trees derived from both typing schemes were compared [22]. The analysis included 136 sequences from isolates in this study for which both MLST profiles and *ompA* genes were successfully amplified, after removal of duplicate sequences with identical profiles. In addition, 42 *C. pecorum* strains with publicly available MLST and *ompA* data were retrieved from the NCBI database.

Maximum likelihood phylogenetic trees were constructed using the GTR+G+I model in MEGA11 [17], with bootstrap support calculated from 1000 replicates. To evaluate the degree of concordance between the two phylogenies, a tanglegram and a strict consensus tree were generated using Dendroscope 3 [23], enabling visual comparison of topological relationships between MLST- and *ompA*-based reconstructions. To quantify the incongruence between the two evolutionary relationships, we calculated the Robinson–Foulds distance using the appropriate R package (R software, version 4.5.1) [24].

## 3. Results

### 3.1. Epidemiological Characteristics of C. pecorum Infections

In this study, *C. pecorum* was the only *Chlamydia* species detected, with 250 positive cases identified, corresponding to an overall infection rate of 18.91% (250/1322). The prevalence and molecular characteristics of *C. pecorum* detected from bovine rectal swabs across multiple provinces are summarized in Table 1. Detection rates varied markedly among regions and cattle types. The highest prevalence was observed in beef cattle from Wuzhong, Ningxia (55.56%, 45/81), followed by adult Holstein cows in the same region (48.62%, 88/181). In contrast, no infections were detected in Holstein Cow (Calf) (0/36). The lowest prevalence was recorded in Simmental beef cattle from Jin-chang, Gansu Province (1.85%, 2/108).

### 3.2. Molecular Typing and Evolutionary Characteristics of C. pecorum ompA

Cluster analysis of the *ompA* gene revealed 73 distinct alleles among the 246 sequences obtained. Sequence alignment showed that the intraspecific similarity of *ompA* among domestic strains ranged from 73.37% to 100%, while similarity to international sequences from GenBank ranged from 72.41% to 100%. These findings indicate that *C. pecorum* strains in China display genetic diversity comparable to global isolates.

**Figure 2:**
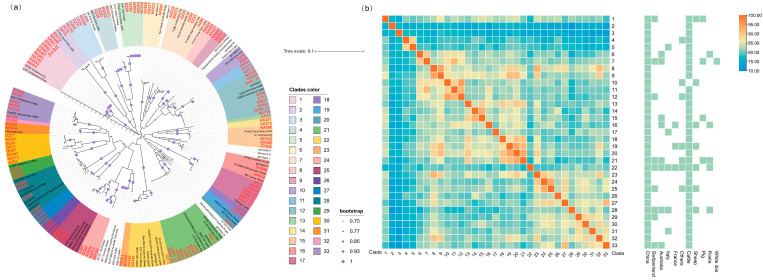
Neighbor-joining (NJ) phylogenetic analysis of *C. pecorum* based on the variable domains (VDs) of the *ompA* gene. (**a**) Phylogenetic tree constructed from 800 bp fragments of 132 strains identified in this study (highlighted in bold red) and 74 reference sequences obtained from GenBank. Thirty-three clades were identified, each indicated by a distinct color. (**b**) This heatmap illustrates the genetic divergence across 33 clades as a symmetrical matrix, with rows and columns identically ordered. Annotation strips on the right mark the associated geographic and host distribution of *C. pecorum* for each clade.

Several unique phylogenetic clusters were identified in western China that have not been reported previously. For example, Clades 3, 24, and 30 were detected in more than one province, suggesting inter-provincial transmission and underscoring the need for monitoring the potential spread of these lineages (Figure 2a). Region-specific clusters were also observed in Ningxia, Tibet, and Qinghai. In Tibet, two clusters were detected: Clade 18 in yaks and Clade 10 in goats, the latter showing close relatedness to strains from Morocco. In Ningxia, seven unique clusters were identified, which demonstrated greater genetic diversity than those from the three other provinces.

The heatmap further illustrated the genetic distances among clades, their phylogenetic relationships, and patterns of interspecies transmission and global distribution (Figure 2b). Overall, *C. pecorum* strains from China formed multiple clusters, several of which were unique to the country, highlighting high genetic diversity and local evolutionary adaptation. Clades 2 and 3, which were phylogenetically distinct yet closely related to each other, were detected across multiple provinces, suggesting strong dissemination potential and warranting close attention to their pathogenicity and zoonotic risk. Additionally, multiple clades were identified in three or more countries across different continents, pointing to possible intercontinental transmission. Several clusters (e.g., Clades 1, 7, 16, 21, 22, and 28) were also detected in three or more mammalian species, indicating broad host adaptability. These findings demonstrate that strains circulating in western China include lineages with enhanced transmissibility and elevated zoonotic potential, reinforcing the need for intensified surveillance.

### 3.3. C. pecorum MLST Analyses

MLST identified 114 distinct STs of *C. pecorum*. Among these, three (ST289, ST336, and ST338) had been reported previously, while 111 were novel STs documented here for the first time. Of these novel STs, 71 were generated through recombination events involving the seven housekeeping genes, whereas 40 resulted from allelic mutations at one or two loci. All novel STs have been deposited in the MLST database and assigned official ST numbers (Supplementary Materials S3; new allele numbers are indicated in red font). Globally, only 83 STs of *C. pecorum* had been reported prior to this study [25]. Sequence alignment revealed high genetic similarity among STs, ranging from 99.06% to 99.97%. These findings underscore the remarkable genetic diversity of *C. pecorum* in western China, which exceeds previously documented levels, and highlight the need for closer attention to its potential impact on human and animal health in this region.

**Figure 3:**
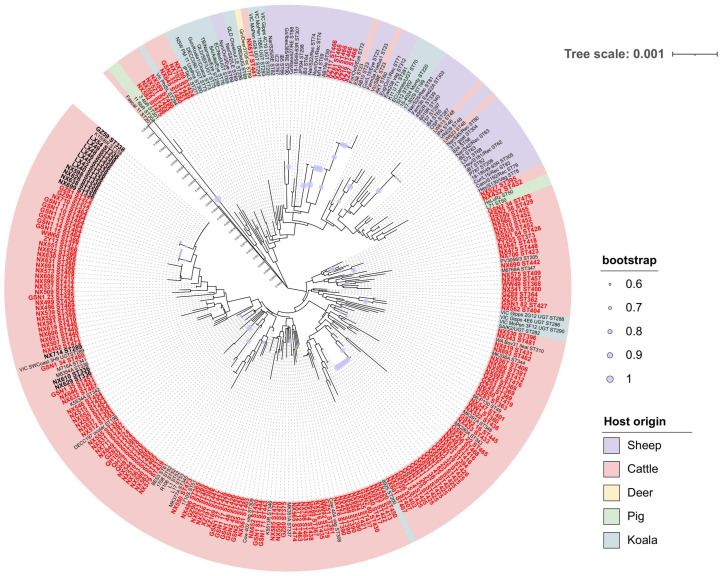
Phylogenetic analysis of *C. pecorum* based on multilocus sequence typing (MLST). Sequence types (STs) identified in this study are highlighted in enlarged bold font: black indicates previously reported STs, while red denotes novel STs identified here. The phylogenetic tree is color-coded by host origin, with five categories represented: cattle, sheep, koalas, pigs, and deer.

Given their concordance with whole-genome and core-genome phylogenies, concatenated MLST sequences were used to infer phylogenetic relationships; host origins were then indicated by color coding. Phylogenetic analysis showed that strains from western China formed multiple unique clades distinct from known global lineages, reflecting both high diversity and unique evolutionary trajectories (Figure 3). Comparative analysis of ST distribution across porcine, bovine, ovine, koala, and deer hosts revealed host-specific evolutionary patterns. Notably, cross-host transmission was evident: koala- and bovine-associated STs (e.g., ST48, ST23) were detected in ovine lineages, while ovine- and bovine-associated STs (e.g., ST69) were found in koala clusters. The highest diversity and most complex phylogenetic relationships were observed in cattle, suggesting that they may serve as reservoir hosts for multiple genotypes. Given the economic importance of cattle and their close contact with humans, these findings raise concern regarding zoonotic transmission through direct contact or consumption of dairy products.

**Figure 4:**
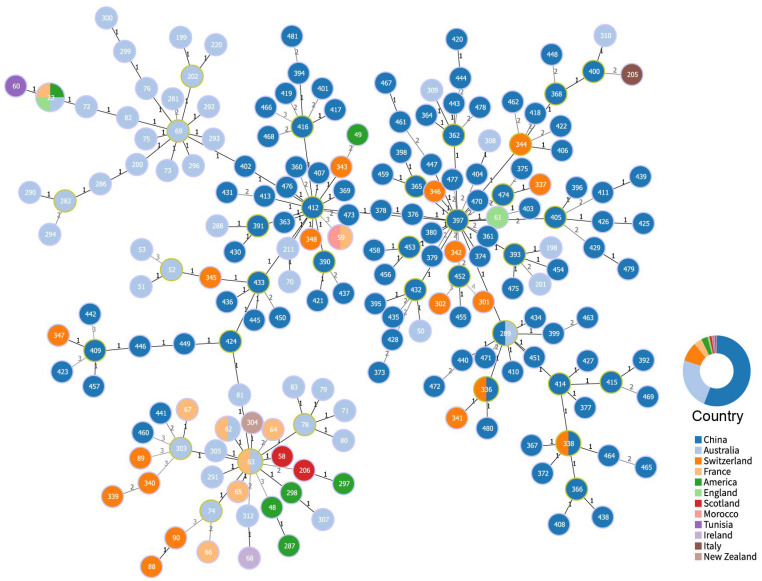
Cluster analysis of *C. pecorum* sequence types (STs). Numbers on connecting lines indicate allelic (locus) differences between nodes. Clonal complexes (CCs) defined by the double-locus variant (DLV) criterion are highlighted with yellow circles. Nodes are labeled with their corresponding STs and color-coded by country of origin. The concentric circle chart depicts the percentage distribution of STs within each country.

A minimum spanning tree (MST) analysis was conducted on 203 sequences grouped by country (Figure 4). goeBURST analysis based on MLST allelic profiles identified 194 unique STs, of which 111 originated from western China. Using a DLV threshold, these STs were clustered into 33 CCs. Within western China, 24 CCs were detected, 21 of which were unique to China, further highlighting the exceptional genetic diversity of local strains. In contrast, ST338 and ST336 CCs were shared with Switzerland, and ST289 with Australia, indicating greater adaptability and potential for international dissemination of these lineages. Among the Chinese CCs, ST412 and ST397 were predominant, each comprising multiple isolates and inferred to represent ancestral genotypes with high clonal relatedness. Minimum-evolution phylogenetic analysis revealed a distinct evolutionary trend among *C. pecorum* CCs in western China, underscoring the need for further investigation into the virulence and transmission capacity of these lineages.

### 3.4. MLST and ompA Phylogenetic Tanglegram

**Figure 5:**
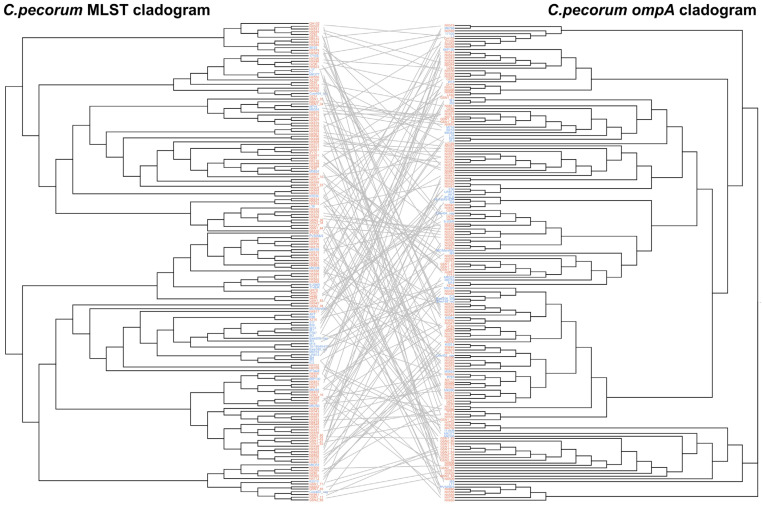
Tanglegram comparing MLST- and *ompA*-based phylogenies of *C. pecorum*. Midpoint-rooted phylogenetic trees were constructed using the maximum likelihood method and reconciled to assess topological congruence. The analysis included 136 sequences obtained in this study and 42 reference sequences from GenBank. Phylogenetic tree showing *C. pecorum*: red font for those identified in this study; blue font for reference strains from GenBank, with their respective names provided at the leaf of the tree.

To further evaluate phylogenetic relationships within *C. pecorum*, a tanglegram was constructed to compare evolutionary placements derived from MLST and *ompA* trees (Figure 5). Phylogenetic analysis revealed significant topological incongruence between the two trees, visually manifested by numerous crossing connections. This visual assessment was supported by quantitative data: the calculated Robinson-Foulds distance was 282 (normalized value: 0.194), indicating a substantial divergence between the evolutionary history of the *ompA* gene and that of the core genome-based MLST framework. Notably, strains belonging to the same ST did not consistently cluster within the same branch of the *ompA* tree, while strains with identical *ompA* sequences were often distributed across separate branches of the MLST tree. Although both methods demonstrated considerable genetic diversity, the observed discordance highlights the limitations of current typing approaches in fully resolving the ecology and evolutionary dynamics of *C. pecorum*.

## 4. Discussion

A total of 1322 swab samples were collected from livestock in western China, of which 250 (18.91%) were positive for *C. pecorum*. Both *ompA* and MLST analyses revealed substantial genetic diversity among the *C. pecorum* from western China. Several unique *ompA* genotypes were identified as being prevalent specifically in western China, and 111 novel STs were reported here for the first time. Together, these findings demonstrate that *C. pecorum* in this region consists of multiple distinct phylogenetic clusters and displays remarkable genetic heterogeneity. In light of recent human infections in the same region, this observed diversity raises a concrete concern: a broader genetic pool increases the potential for the emergence of variants with enhanced adaptability to human hosts. Our findings therefore move beyond documenting diversity to highlighting a potential source for zoonotic emergence, underscoring the need for strengthened surveillance and risk assessment.

In this study, we investigated the prevalence of *Chlamydia* in 1322 rectal swab samples collected from cattle across seven regions in four Chinese provinces, with an overall infection rate of 18.91%. All positive samples were identified as *C. pecorum*, which contrasts with previous reports documenting *C. abortus*, *C. pneumoniae*, and *C. psittaci* in cattle [26]. The exclusive detection of *C. pecorum* in our study may reflect the use of rectal swabs as the sole sampling method, which could bias detection toward intestinally localized species. To provide a more comprehensive understanding of Chlamydial infections in livestock, future studies should include samples from multiple anatomical sites in both cattle and sheep.

It is well established that *ompA* is among the most genetically diverse markers within the genus *Chlamydia*, and its characterization is critical for the development of MOMP-based vaccines and serological assays [10]. In this study, phylogenetic analysis of *ompA* sequences demonstrated that, even at a 96% similarity threshold, *C. pecorum* in western China could be divided into 33 clades, several of which exhibited evidence of cross-host transmission and intercontinental spread. Compared with previous reports, Chinese strains displayed greater diversity, characterized by multiple independent evolutionary branches and the coexistence of distinct clades within a single province, a phenomenon rarely described in earlier studies [6,10]. The observed patterns of cross-host transmission and intercontinental spread, suggest that both long-range animal movement and local wildlife-livestock interface interactions are drivers shaping the genetic diversity [2,27]. This extensive heterogeneity underscores the challenges in designing broadly effective MOMP-based vaccines and diagnostics, while simultaneously highlighting the significant and evolving public health threat posed by this pathogen.

Consistent with earlier findings, the high variability of *ompA* limits its value as a standalone marker for inferring genome-wide phylogeny. In contrast, MLST provided high-resolution genetic discrimination, enabling the tracking of emerging clonal lineages and revealing broader patterns of *C. pecorum* circulation between wild-life and livestock [13,28]. The discovery of 111 novel STs among the 182 identified provides compelling evidence for unique evolutionary events within the region. The fact that a majority of these novel STs (71/111) originated from recombination in housekeeping genes implies that inter-strain genetic recombination may be a mechanism driving the evolution and diversification of *C. pecorum* in western China [29]. Furthermore, the emergence of the remaining novel STs through allelic mutations points to the additional role of local adaptation and selective pressures, potentially fueled by geographical isolation and distinct host environments [30]. While MLST phylogenetic analyses confirm that cross-host transmission and intercontinental spread persist across ST lineages, the overwhelming predominance of novel, geographically restricted STs suggests that local evolutionary forces currently outweigh the impact of introduction events.

The genome of *C. pecorum* is highly plastic, exhibiting marked diversity in *ompA* sequences and STs, with phylogenetic analyses revealing extensive cross-branching between different genetic markers—a pattern consistent with homologous recombination [22]. Indeed, interclade recombination has been demonstrated as a key mechanism for the spread of genomic islands, such as the tetracycline resistance element, in *C. suis* [31]. Moreover, the presence of multiple *C. pecorum* types within the same cattle population on a single farm, together with earlier reports of co-infection in different anatomical sites of individual animals [6,26], underscores the complexity of its epidemiology and creates opportunities for such genetic exchange. Additional molecular approaches are therefore needed to enhance genotyping resolution, improve source tracing, and strengthen risk assessment. Importantly, *C. pecorum* can cross host barriers, and co-infections with other *Chlamydia* species are common in wildlife, for example, *C. pneumoniae* in koalas and the zoonotic *C. abortus* in sheep [32,33]. These multi-species infections provide a plausible setting for lateral gene transfer, a process that is known to occur among *Chlamydia* species and can involve plasmid exchange and recombination [27,34]. Thus, the possibility of genetic exchange between *C. pecorum* and other chlamydiae during co-infection cannot be excluded [35]. With intensifying human activities and global exchange, the risk of cross-species transmission and further genetic diversification of *C. pecorum* is increasing, underscoring the need for vigilant surveillance of its zoonotic potential [36].

The growing recognition of the threat posed by *Chlamydia* species to human health, which contributes to a significant global disease burden [37,38], is compounded by rising concern over the zoonotic potential of *C. pecorum*, highlighting the need for enhanced risk assessment and vigilance [4]. Advances in diagnostic technologies have also led to the reclassification of species once considered non-zoonotic, such as *C. caviae* and avian *C. abortus* strains, as zoonotic agents, following an increasing number of confirmed human infections [39,40]. At the same time, the widespread application of molecular detection methods has revealed a significant rise in the prevalence of *C. psittaci*, a long-recognized zoonotic pathogen [41]. In China, the first globally documented case of human infection with *C. pecorum* was reported in 2022, presenting as severe pneumonia, followed by a second fatal case in 2023, which attracted international attention [8,9]. Both cases were confirmed by mNGS. These reports suggest that human infections with *C. pecorum* are likely underdiagnosed, owing to limited clinical awareness of its zoonotic potential and restricted access to mNGS in remote, livestock-dependent regions.

Nonetheless, ethical restrictions prevented us from sampling farm workers in this current study to directly assess infection status among individuals with frequent livestock exposure. To proactively address this critical knowledge gap and directly assess the spillover risk, we have established multiple monitoring and control sites across western China. This surveillance network is uniquely designed to collect and analyze samples from human, animal, and environmental sources.

## 5. Conclusions

In summary, our study demonstrates that *C. pecorum* is widely distributed in western China and exhibits remarkable genetic diversity, as evidenced by the divergence of *ompA* sequences into multiple phylogenetic clusters and the identification of a large number of novel STs by MLST analysis. These findings significantly enhance our understanding of the genetic characteristics and epidemiological patterns in this region. This work provides an essential genetic database and a solid scientific foundation for future source-tracing during outbreak investigations, refined risk assessments, and the formulation of targeted control strategies. Critically, in direct response to the zoonotic threat identified, we have established a multi-site surveillance network that includes human, animal, and environmental monitoring. This system is poised to enable the timely detection of human infections and isolation of pathogenic strains, thereby creating a critical early-warning and research resource to mitigate the public health risk posed by this emerging pathogen.

## Figures and Tables

**Table 1:** Prevalence of *C. pecorum* of Livestock from provinces of China.

Province	City/Prefecture	Host Species	Positivity
Gansu Province	Zhangye	Simmental Beef Cattle	16.34% (75/459)
	Wuwei	Simmental Beef Cattle	6.80% (8/103)
	Linxia	Simmental Beef Cattle	33.33% (12/36)
	Jinchang	Simmental Beef Cattle	1.85% (2/108)
Qinghai Province	Yushu	Yak	12.00% (6/50)
Ningxia	Wuzhong	Holstein Cow (Adult)	48.62% (88/181)
	Wuzhong	Holstein Cow (Calf)	0.00% (0/36)
	Wuzhong	Beef Cattle	55.56% (45/81)
Tibet	Ngari	Goat	4.83% (7/145)
	Ngari	Yak	5.69% (7/123)

## Data Availability

The data supporting the findings of this study are available in the following repositories: ompA gene nucleotide sequences: GenBank (accession numbers are listed in Supplementary Materials S2) [https://www.ncbi.nlm.nih.gov/, accessed on 10 September 2025]. Multilocus Sequence Typing (MLST) profiles: PubMLST database for Chlamydiales [http://pubmlst.org/chlamydiales/, accessed on 15 August 2025]. Genotype IDs are provided in Supplementary Materials S3.

## Supplementary Materials

The following supporting information can be downloaded at: https://www.mdpi.com/article/10.3390/pathogens14121209/s1.
