# Supplementary material for: Epidemiology and Genetic Diversity of Chlamydia pecorum in Cattle and Sheep from Western China
Source: Pathogens. 2025 Nov 27;14(12):1209. doi: 10.3390/pathogens14121209 (PMC12735864; doi:10.3390/pathogens14121209)
Supplement: Supplementary file 1 [file pathogens-14-01209-s001.zip › Supplementary material S1.pdf]

Table S1 Primers used for *ompA* in the present study

Gene	Primer	Sequence (5′ - 3′)	Annealing temp (°C)	Amplicon size (bp)
ompA	CMGP-1F	CCTTGTGATCCTTGCGCTACTTG	50℃	800bp
	CMGP-1R	GTGAGCAGCTCTTTCGTTGAT		
	CMGP-2F	GTAACGCCTCCTTACACGCTTTG		
	CMGP-2R	GTAAAGTTGCTCCCATGGAAACAC		

Note: 1F: First round upstream primers; 1R: First round of downstream primers; 2F: Second round of upstream primers; 2R: Downstream products of the second round
